# Macro-level Modeling of the Response of *C. elegans* Reproduction to Chronic Heat Stress

**DOI:** 10.1371/journal.pcbi.1002338

**Published:** 2012-01-26

**Authors:** Patrick D. McMullen, Erin Z. Aprison, Peter B. Winter, Luis A. N. Amaral, Richard I. Morimoto, Ilya Ruvinsky

**Affiliations:** 1 Department of Chemical and Biological Engineering, Northwestern University, Evanston, Illinois, United States of America; 2 Department of Ecology and Evolution, Institute for Genomics and Systems Biology, The University of Chicago, Chicago, Illinois, United States of America; 3 Department of Molecular Biosciences, Rice Institute for Biomedical Sciences, Northwestern University, Evanston, Illinois, United States of America; Indiana University, United States of America

## Abstract

A major goal of systems biology is to understand how organism-level behavior arises from a myriad of molecular interactions. Often this involves complex sets of rules describing interactions among a large number of components. As an alternative, we have developed a simple, macro-level model to describe how chronic temperature stress affects reproduction in *C. elegans*. Our approach uses fundamental engineering principles, together with a limited set of experimentally derived facts, and provides quantitatively accurate predictions of performance under a range of physiologically relevant conditions. We generated detailed time-resolved experimental data to evaluate the ability of our model to describe the dynamics of *C. elegans* reproduction. We find considerable heterogeneity in responses of individual animals to heat stress, which can be understood as modulation of a few processes and may represent a strategy for coping with the ever-changing environment. Our experimental results and model provide quantitative insight into the breakdown of a robust biological system under stress and suggest, surprisingly, that the behavior of complex biological systems may be determined by a small number of key components.

## Author Summary

Dynamic response to changing conditions in the environment is an essential property of all biological systems. Whereas extensive research over the last several decades has elucidated numerous molecular responses to environmental stress, there is much less known how these translate into organismal-level responses. Two types of modeling approaches are often used to bridge this gap. Fine-grained models seek to explain phenomena as resulting from interactions of large numbers of individual components. This approach demands a highly detailed knowledge of the underlying molecular mechanisms and has an inherent difficulty in crossing spatial scales and organizational hierarchies. As an alternative, here we present a macro-level model of reproduction in *C. elegans* that uses fundamental engineering principles, together with a limited set of experimentally derived facts, to provide quantitatively accurate predictions of performance under a range of physiologically relevant conditions. One important finding is that individuals within a population display considerable heterogeneity in their response to heat stress. This could be a reflection of different strategies for coping with the ever-changing environment. Our study further demonstrates that dynamic behaviors of systems may be determined by a small number of key components that lead to the emergence of organismal phenomena.

## Introduction

Much of modern biology is inherently reductionist, seeking to enumerate interactions and components to elucidate the inner workings of cells and organisms. However, phenotypes often cannot be explained simply as the sum of the properties of the micro-components. Emergent phenomena [1] are not unique to biology; physical [2], [3], [4], chemical [5], and social [6], [7], [8], [9] systems all have to contend with this challenge.

Over the last several decades, thousands of studies have employed genetic and biochemical approaches to reveal the components of biological processes. High-throughput technologies have greatly accelerated discovery, generating detailed parts lists for cellular systems [10], [11], [12]. Such abundance of data facilitated development of fine-grained models that provided quantitatively accurate descriptions of signaling [13], transcriptional regulation [14], and the heat shock response [15].

Despite the success of this general approach, it cannot be used in circumstances when detailed understanding of molecules and processes is not available. While this limitation can be overcome by additional experimentation, fine-grained models have an intrinsic difficulty in connecting cellular phenomena to organismal behavior [1], [16], [17], [18], [19]. An alternative is to use macro-level modeling, which although omitting many specific details, could if properly constructed, describe the overall performance of complex systems [20], [21], [22].

Due to its easily quantifiable output, the reproductive system offers an attractive opportunity to bridge the molecular biology of a process and the emergence of dynamic, organismal-level phenotypes. Reproduction in *Caenorhabditis elegans* has been extensively studied using genetic [23], [24], [25], [26], [27], [28] and biochemical [29], [30], [31], [32], [33] approaches. *C. elegans* hermaphrodites are self-fertile [34]. They first produce a finite cache of sperm [35], and then irreversibly transition to oocyte production [36], [37], [38], which occurs continuously until reproductive senescence [39]. The overall reproductive output is primarily determined by the availability of sperm [34], [40], because their number is set for the lifetime of an individual. Many of the specific molecular components involved in gametogenesis and later reproductive events have been characterized [41], [42], [43], [44], [45], [46], [47]. For example, a signaling mechanism directly couples oocyte maturation and ovulation to the presence of sperm [31], [32], [48].

Although considerable information is available about the components of the reproductive system, we are interested not in specific molecular interactions, but rather in understanding how individual animals reproduce. The distinction between these two questions can be compared to the difference between studying the molecular biology of neurons and human behavior [17]. Our goal here is to construct a parsimonious macro-scale model that is grounded in experimental data. If such a model could provide quantitatively accurate predictions, it would serve to identify a minimal set of biological components and processes necessary to endow the reproductive system with its characteristic dynamics.

A time-tested approach to investigating macro-level processes is to perturb the environment in a controlled way and to measure the system's subsequent response. Temperature has often been used to probe dynamic behavior, as well as components and organization of biological systems [49], [50], [51]. This is because organisms are sensitive to environmental conditions and because temperature can be easily and precisely manipulated in the laboratory setting. Here, we analyzed the effects of chronic elevated temperatures on *C. elegans* reproduction to connect molecular processes to macroscopic phenotypes, particularly those involved in dynamic responses of organisms to a changing environment.

## Results

We sought to ensure that our model of *C. elegans* reproduction was biologically reasonable. Because sufficiently detailed experimental data were not available, we first collected extensive, time-resolved datasets on egg-laying performance under a variety of temperature regimes. Next we formulated a parsimonious model that incorporated several key elements of the reproductive system that were previously described in the literature and trained our model using a subset of the collected experimental data. Finally, we tested the performance of the model under different environmental conditions and in different genetic backgrounds.

### *C. elegans* reproduction is exquisitely sensitive to temperature changes

**Figure 1:**
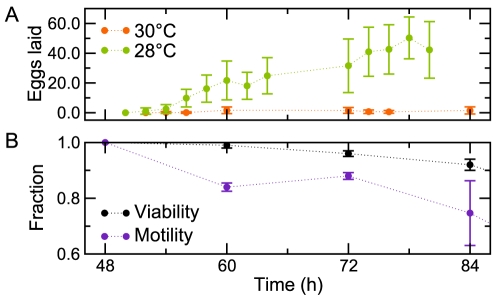
Reproduction is sensitive to chronic temperature changes. The average number of eggs laid by an individual hermaphrodite is substantially lower at 28°C (compared to ∼300 at 20°C), and is nearly zero at 30°C (A). In contrast, at 30°C, animals exhibit considerably milder effects on motility and viability (B).

Compared to the well-understood heat shock response, less is known about how organisms respond to chronic, moderate temperature stress. It is well established that the average number of eggs laid by *C. elegans* hermaphrodites is dependent on temperature [35]. We asked whether reproduction is more temperature sensitive than other vital processes and how *individual* worms respond to temperature stress. We examined viability, movement, and reproductive output over a range of temperatures (Table 1, Table S1). We developed an experimental protocol in which nematodes were reared at the commonly used cultivation temperature of 20°C, and then, just prior to the onset of reproduction, *individually* shifted to various elevated temperatures. This treatment—chronically exposing worms to temperatures between 20°C and 30°C—is *qualitatively* different from the standard acute heat shock experiments, which involve brief exposure to nearly fatal temperatures (33°C) [52]. Whereas the average number of eggs laid at 28°C was substantially reduced compared to temperatures at which worms are routinely raised (see below), at 30°C reproduction ceased completely (Figure 1A). In contrast, neither viability nor motility was comparably affected (Figure 1B).

**Table 1:** Experiments performed to determine the dynamics of *C. elegans* reproductive behavior.

Temperature (°C)	Independent Experiments	Nematodes Assayed	Eggs Counted
20	8	569	40,099
23	3	448	27,137
25	8	491	48,395
28	6	903	20,761
29	7	873	7540
30	2	197	160
	34	3,481	144,092

**Figure 2:**
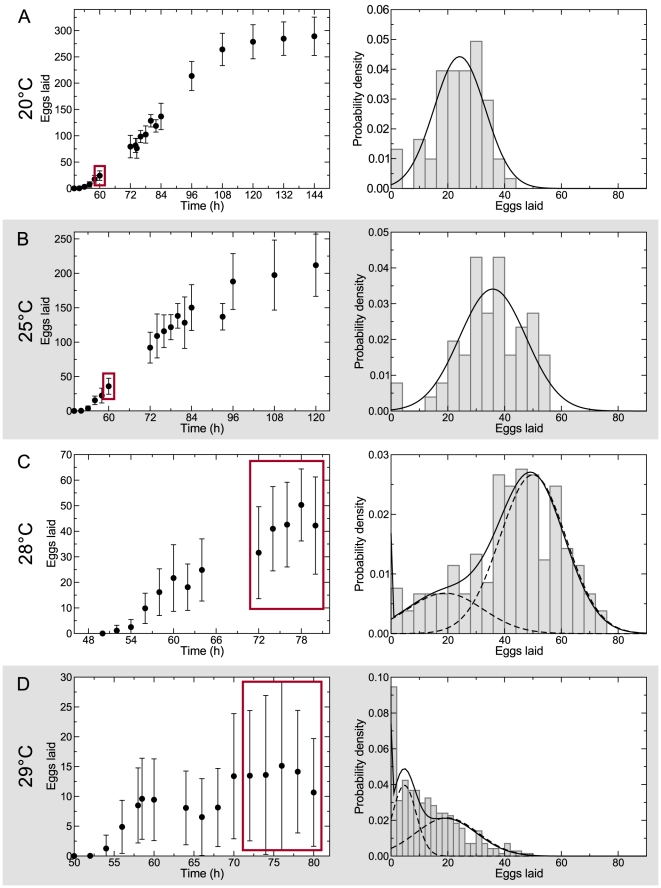
Chronic temperature stress exposes heterogeneous physiological response of the reproductive system in *C. elegans*. The brood sizes for animals reproducing at 20 (A) and 25°C (B) are normally distributed. However, at higher temperatures, 28 (C) and 29°C (D), the distribution of brood sizes reflects a heterogeneous population. At these temperatures, the brood size distributions (solid lines) can no longer be approximated as single normal distributions. Instead, each is better explained as a mixture of two distinct components (dashed lines), the relative weight of which is dependent on temperature. Red boxes in the left panels highlight the data shown in the right panels.

We documented the reproductive performance of 3,418 individual worms, which laid a total of 144,092 embryos (Table 1, Figure S1, Text S1). Importantly, we collected dynamic, time-resolved egg-laying curves, not simply overall brood sizes. The temperatures used in our studies (20–30°C) are likely to be physiologically relevant because *C. elegans* have been isolated from tropical and equatorial locales [53], [54] where temperatures routinely exceed 30°C. Furthermore, nematodes appear to dwell in compost and rotting vegetable matter [55], [56], where temperatures can be even higher than in the ambient environment [57]. Brood size of animals cultivated at 20 and 25°C were normally distributed (Figures 2A, B, S2, S3, Text S1). While the means of the brood size distributions varied with temperature, they had indistinguishable coefficients of variation (*p* = 0.58±0.01, permutation test). These results suggest that while the mean output of the reproductive system is temperature-dependent, increasing temperature does not lead to an appreciable increase in the individual-to-individual variability (Figure S4).

At 28°C, however, we observed a qualitatively different behavior—there were more individuals laying low numbers of eggs than would be expected from a normally distributed population (Figure 2C). This was accompanied by a coefficient of variation (Figure S4) that was significantly higher at 28°C than at 25°C (*p* = 2×10^−4^, permutation test). Furthermore, these data could not be captured by a single normal distribution (p<10^−4^, Kolmogorov-Smirnov test), but could be well described by a mixture of two distributions (Figure 2C). The relative proportion of animals laying a lower than expected number of eggs increased at higher temperatures (Figure 2D), as evidenced by the increase in the coefficient of variation (Figure S4). These results suggest that whereas across a range of lower temperatures reproductive systems of all worms are robust, at higher temperatures, only a fraction of individuals continue to act in a robust manner, *revealing an inherent heterogeneity in physiological response*.

### Simple macro-level model closely reproduces experimental results

**Figure 3:**
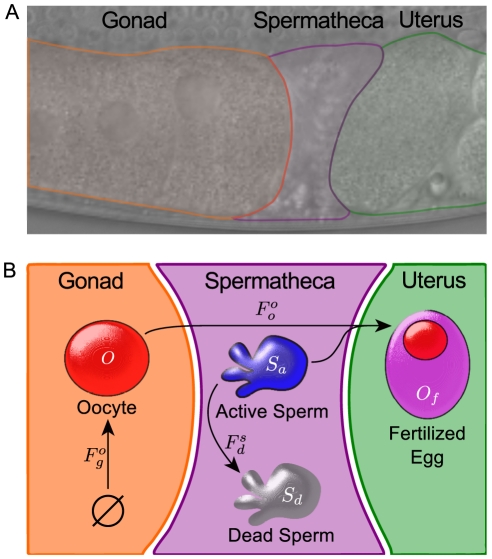
Modeling the dynamics of *C. elegans* reproduction. The reproductive system of a hermaphrodite consists principally of three compartments: the gonad, spermatheca, and uterus (A). The model tracks gametes through these compartments according to mass-action kinetics and parsimonious biological rules (B).

We developed a macro-level model of the *C. elegans* reproductive system. Our model is both simple (it includes a small set of essential features and parameters) and falsifiable (designed to be experimentally testable). The reproductive system (Figure 3A) can be abstracted as a pipeline for the serial maturation and subsequent fertilization of oocytes. We conceptualized it as a series of interconnected compartments—the gonad (which is encapsulated by the gonadal sheath), spermatheca, and uterus—through which gametes flow (Figure 3B). This process can be likened to a chemical reaction because transitions between compartments can be modeled as the conversion of precursors to products. We made two simple but plausible assumptions (a list of major model assumptions is given in Table 2). First, all gametes in the model are conserved and can be explicitly accounted for [58]. Second, all transitions between states obey mass-action kinetics. The latter is a typical assumption for dynamic systems, used in analysis of chemical reaction kinetics [59]. It states that a process proceeds at a rate that is proportional to the availability of each of its inputs.

**Table 2:** Major assumptions of the model.

1.	All gametes in the model are conserved and can be explicitly accounted for.
2.	All transitions between states obey mass-action kinetics.
3.	Oocytes are generated at a constant rate, subject to saturation that prevents *O* from increasing beyond an upper limit established by gonad size.
4.	Chronic exposure to higher temperatures results in gamete death.
5.	*k* ^max^ varies between individuals and is drawn from a normal distribution.
6.	The number of sperm and the timing of the onset of embryo production are determined by the same variable drawn from a normal distribution.
7.	*k_o_*, *k_d_*, and *δ* have an exponential dependence on temperature.

Although oocyte development and maturation involves a number of discrete steps and processes [48], [60], [61], for simplicity, we subsume them into a single state. This mathematical abstraction simplifies the subsequent calculations and reflects the difference between a fine-grained molecular model and a macro-level approach. We represent the number of oocytes, that are generated de novo, as *O*. Experimental data suggest that the total number of germ cells in adults [62] and the rate of oocyte production [48] are constant. Therefore our model treats the rate at which oocytes are generated as a constant, subject to saturation that prevents *O* from increasing beyond an upper limit established by gonad size [48]. Together, these assumptions define the rate 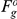 of oocyte creation (Figure 3B),
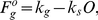
(1)where *k_g_* is a rate constant describing the generation of *O*, and *k_s_* is a rate constant pertaining to the carrying capacity of the gonad.

Hermaphrodites of the standard laboratory strain (Bristol or N2) of *C. elegans* produce approximately 300 sperm during development before the germline irreversibly transitions to oogenesis [34]. Because animals produce oocytes continuously until their cache of sperm is depleted, the number of sperm determines the overall fecundity [34]. A dedicated mechanism communicates the presence of sperm to the developing oocytes. Sperm release major sperm protein (MSP) into the proximal gonad [63], where it induces meiotic maturation of the proximal oocyte [31], [48]. Concomitantly, MSP promotes sheath cell contraction, leading to ovulation [32]. As the oocyte is pulled into the spermatheca, fertilization takes place [64]. After the spermatheca, the embryo passes to the uterus where it completes the first several cell divisions before being laid [24]. The dynamics of egg-laying are known to be bursty, but the time intervals between these bursts are typically on the order of minutes [65], much shorter than the time intervals at which we counted eggs. Therefore we need not consider these dynamics in our model.

The reproductive rate, while approximately constant early in adulthood, decreases as the animals age [66]. This decline in reproductive function likely has multiple causes. In the first several days of reproductive maturity it likely reflects the decreasing number of sperm and the coupling of ovulation to sperm number [63], because mating during this period can produce substantially more progeny [67], [68]. About 5 days after the onset on reproduction, oocyte quality becomes compromised [69], [70], and mating of week-old hermaphrodites does not increase their brood size [68]. At lower temperatures (e.g., 20°C), within 4–5 days of reproductive maturity nearly all of a hermaphrodite's sperm have been used to fertilize eggs [34]. However, it is reasonable to expect that chronic exposure to higher temperatures will result in gamete death. While developing oocytes are likely damaged by chronic temperature stress, they can be continuously generated, therefore their destruction is difficult to decouple from a decrease in their production rate. We thus captured this process by allowing net oocyte production rate in the model to vary with temperature. These assumptions, and their related mass action kinetics, yield expressions for the rate of ovulation 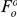 and the rate of sperm death 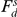,
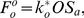
(2a)

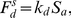
(2b)where *S_a_* is the number of active sperm, 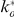 is a rate constant of ovulation, and 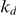 is a rate constant of sperm death.

Because *O* rapidly achieves a steady state [48], we simplified the model specified in Equations 1 and 2 using a quasi-steady-state approximation [71]. We found that this reformulation results in a model that captures the system dynamics equally well (see next section and Text S1). We explicitly solved the steady-state mass balance equation to obtain 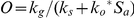 (see Text S1). This allowed us to express the dynamics of the system using a smaller subset of parameters. In the interest of parsimony, we used the parameter *k* ^max^ to summarize the intrinsic maximum rate of oogenesis,
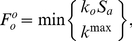
(3)where 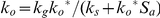 depends weakly on *S_a_*, and can be treated as a constant (see Text S1).

Together, these assumptions can be combined into a system of mass balance equations describing the dynamics of *C. elegans* reproduction,
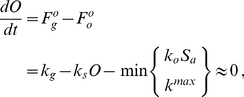
(4a)

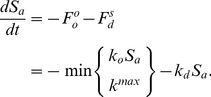
(4b)


In our experiments, we observed substantial variability in both the overall fecundity and the dynamics of egg-laying among individuals. We hypothesized that this variability arises from differences in the intrinsic capacity (*k* ^max^) for oogenesis and the number of sperm produced by each animal, both of which we surmised are normally distributed (Figures 2A, B, S2, S3, Text S1). The rate of sperm production is approximately constant over time [72], and high sperm count is associated with delayed onset of oogenesis [67]. To capture this, when simulating our model, the number of sperm of each individual and the timing of the onset of embryo production were determined by the same variable drawn from a normal distribution.

Recalling the heterogeneity of brood sizes at higher temperatures (Figure 2), we reasoned that the fraction (*δ*) of animals that exhibit a non-robust reproductive output varies with temperature, and treated *δ* as a free parameter. Although the mean-field behavior of our model can be analytically solved (Text S1), we solved it numerically. We used maximum likelihood estimation [73] to determine the kinetic parameters for our model. Interestingly, our estimates of *k* ^max^ were substantially different for the two classes.

**Figure 4:**
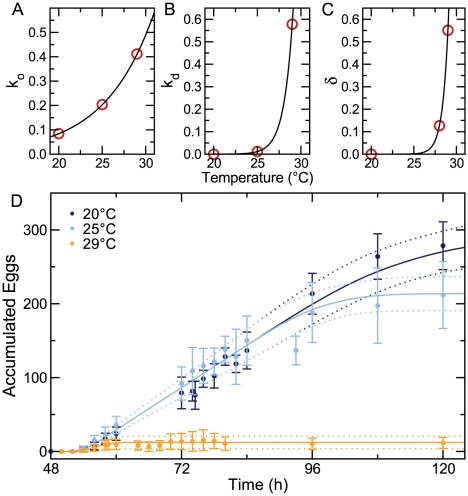
Fitting the model to experimental data. Because the reproductive dynamics are strongly temperature dependent, we let the three model parameters vary as exponential functions of temperature (A–C). As expected, all parameters increased with temperature. Red circles represent the estimated parameters values for the three temperatures used to train the model. Constraining model parameters yielded close fits to experimental observations, represented by dots ±1 standard deviation (D). Model predictions (solid lines) ±1 standard deviation (dashed lines) are shown for comparison.

We used time-resolved, densely sampled egg-laying curves collected at 20, 25, and 29°C (Table 1, Figure 2) to train our model for both the robust and non-robust classes of animals. Noting the narrow range of relevant temperatures, we hypothesized exponential dependence of the model parameters on temperature. Because *δ* is only nonzero at 28°C and above, we used curves collected at 20, 28, and 29°C to estimate its value more robustly. The estimated coefficients of these exponential functions (Figure 4A–C) result in model predictions that closely recapitulate the empirical data (Figure 4D).

### More complicated models do not offer an improved description of the system

**Figure 5:**
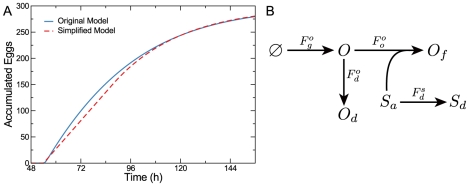
More complicated models do not offer an improved description of the system. Explicitly accounting for oocyte development (blue) is nearly indistinguishable from the quasi-steady-state approximation (red) (A). Including a discrete state for dead oocytes (B) complicates the model, but leads to a description (Equation 6) that is mathematically equivalent to the parsimonious model (Equation 4).

To obtain Equation 3, we surmised that the dynamics of oocyte development are steady-state [48], and the number of developed oocytes *O* is constant (also see Text S1). To ensure that this approximation does not lead to an overly simplistic model that fails to capture aspects of reproductive dynamics, we evaluated predictions for two distinct model formulations. The first assumed that *O* reaches a quasi-steady-state according to Equation 3. This simplified model is fully described in Equation 4. The second was more complicated, explicitly accounting for oocyte generation and development (Equations 1 and 2a) and allowing *O* to vary. Only subtle quantitative differences existed in the predictions of these two models, justifying the use of the parsimonious version (Figure 5A).

To ensure that the parsimonious model (Equation 4) does not omit other details that could improve the description of the system, we constructed an alternative model with an additional component that plausibly exists in the reproductive system: oocyte death. In a model that explicitly included discrete states for dead oocytes (*O_d_*) (Figure 5B), the rate of oocyte accumulation becomes,
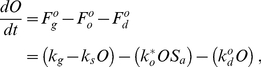
(5)where 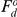 is the rate of oocyte death and 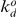 is the rate constant of oocyte death. Reformulating Equation 5, we obtain,
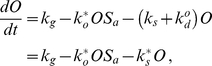
(6)where 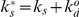. Because this expression is mathematically equivalent to Equation 4a, it is difficult to differentiate between this model that accounts for oocyte death from the more parsimonious model formulated above (Equation 4).

### Testing predictions of the model

**Figure 6:**
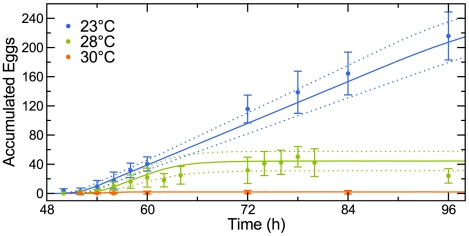
Predicting the dynamics of *C. elegans* reproduction. Predicted egg-laying trajectories (sold lines are median predictions; dashed lines are ±1 standard deviation) for animals shifted to 23, 28, and 30°C quantitatively capture the experimental data (dots; ±1 standard deviation).

Our modeling framework provides the basis for predicting the behavior of animals treated under different conditions and having different genetic backgrounds. As a first test, we generated predictions of the dynamics of reproductive output following chronic temperature shifts conducted under the same experimental protocol that was used to train the model, but at three different temperatures. At 23, 28, and 30°C, we observed a close correspondence between predicted values and experimental results (Figure 6). Predictions were obtained using parameters estimated from the training data (Figure 4); the only additional information that was specified was the temperature to which the animals were exposed. Importantly, in addition to the quantitative matches obtained for the population means, we also observed a correspondence between predicted and experimentally measured animal-to-animal variances of brood sizes.

**Figure 7:**
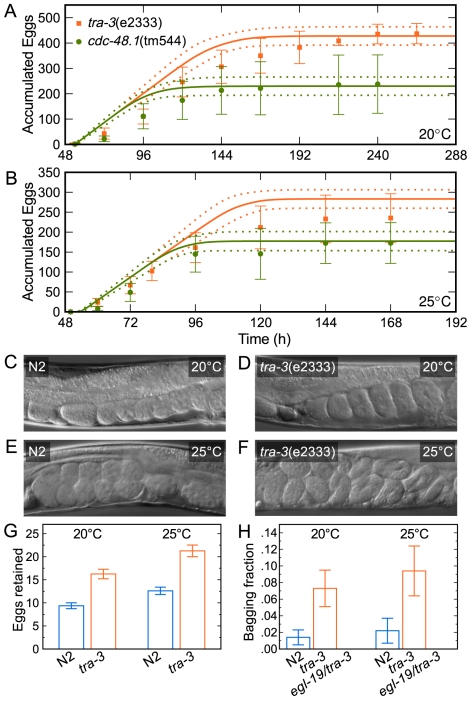
Predicting behavior of *C. elegans* reproductive mutants. The reproductive dynamics of *tra-3* and *cdc-48.1* mutants at 20°C (dots; ±1 standard deviation) are well described by the model (solid lines are median predictions; dashed lines are ±1 standard deviation) (A). At 25°C, *tra-3* animals produce fewer progeny than predicted (B). Embryos are arranged in an orderly fashion in N2 animals at 20 (C) and 25°C (E) and in *tra-3* mutants at 20°C (D), but not at 25°C (F). Consequently, *tra-3* mutants retain more embryos in the uterus than N2 animals (G; average number per worm is shown; ±1 standard deviation). Bagging phenotype of *tra-3* mutants is rescued by an egg-laying constitutive mutation *egl-19*(ad695) (H).

As a second test, we probed the reproductive dynamics of two mutants, *tra-3*(e2333) [74] and *cdc-48.1*(tm544) [75], that produce different numbers of offspring than the wild-type N2 strain (Table S2). In our experimental paradigm, at 20°C these two mutants produced 437±40 and 238±115 progeny, respectively. At least two lines of evidence suggest that availability of sperm is the limiting factor in *C. elegans* reproduction. First, self-fertile hermaphrodites continue laying unfertilized eggs once their cache of sperm becomes exhausted [34], [76]. Second, hermaphrodites that are mated to males generate up to four times the number of progeny as their unmated counterparts because male ejaculate provides many more sperm than the number produced by a hermaphrodite [67], [77]. Relevantly, the *cdc-48.1*(tm544) mutant animals lay approximately as many eggs as the wild type, but a substantial fraction of these oocytes are not fertilized [75]. We therefore reasoned that the number of progeny of individual animals accurately reflected the number of sperm they produced. Using these inferred sperm counts and the model parameters estimated from the training data (Figure 4), we predicted the dynamics of the reproductive output of the two mutants. At 20 and 25°C, predictions for the *cdc-48.1* mutants matched the experimental results, as did predictions for the *tra-3* animals at 20°C (Figures 7A, B). At 25°C, however, the *tra-3* mutants laid fewer embryos than predicted by our model (Figure 7B).

We investigated the plausible causes of this discrepancy. At 20°C the embryos of both the wild-type N2 and *tra-3* animals were arranged in an orderly fashion within the uterus (Figure 7C, D). At 25°C (Figure 7E) the embryos in wild-type animals were more numerous than at 20°C, but this effect was far more pronounced in the *tra-3* mutants, which had retained embryos that were older than the age at which they are typically laid (Figure 7F). The number of embryos retained by individuals correlated with the sperm count, such that retention in the *tra-3* animals was substantially higher than in the wild-type (Figure 7G). We interpreted this as an indication that our model over-predicted the number of eggs laid because it did not consider the accumulation of eggs in the uterus and its possible consequences. The total number of eggs laid and retained in the uterus of the *tra-3* animals at 25°C was indistinguishable from that in the wild-type N2 animals under the same conditions. In contrast, at 20°C *tra-3* mutants produced nearly 50% more offspring (437 vs. 302) reflecting a greater number of sperm. Together, these results suggest that a higher aggregate egg production rate at 25°C results in higher egg retention which causes a mechanical impediment to the passage of eggs and therefore disrupts reproduction.

The accumulation of embryos inside the uterus led to a “bagging” phenotype [78] and eventual hatching within the parent (Figure 7H, Table S3). Significantly, the bagging phenotype of the *tra-3* mutants was completely suppressed by an *egl-19*(ad695) mutation that causes constitutive egg-laying [79]. This suggests that the mechanical elements of the egg-laying apparatus were compromised by chronic heat stress, serving as a physical impediment to achieving the maximum rate of egg-laying and, therefore, the highest brood size given the number of available sperm.

## Discussion

We developed a macro-level, parsimonious model that, although it incorporates only a few of the known elements of the reproductive system of *C. elegans*, is sufficient to make quantitatively accurate predictions of the dynamics of reproduction under stress. Using detailed, time-resolved experimental data, we demonstrated that the model predicts reproductive dynamics of animals in a number of environmental and genetic backgrounds. The molecular details underlying reproduction undoubtedly are numerous and complex. Specifically, large numbers of genes are associated [80] with the following reproduction-related Gene Ontology terms: fertilization (23), oviposition (394), oocyte (60), oogenesis (179), and sperm (52). We have shown that a minimal model of a process can be sufficient for capturing system dynamics. We were able to infer a minimum set of essential elements that are sufficient to describe the temperature-dependent dynamics of reproduction in *C. elegans*.

**Figure 8:**
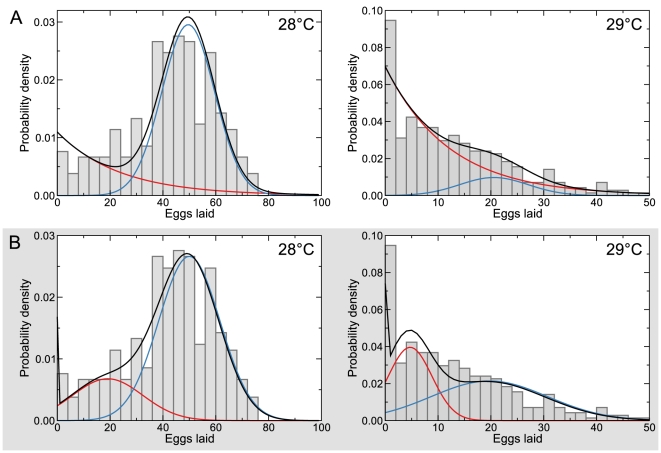
Alternative interpretations of the heterogeneous response to stress by individual nematodes. At permissive temperatures (≤25°C) brood sizes are well described as normal distributions (as shown in Figure 2). However, at higher temperatures (≥28°C), the brood size distributions diverge from normal, and a mixture of two distributions is required to describe the data. Two different combinations of distributions could account for the observations. In both cases a fraction of the overall population consists of worms reproducing robustly; these are described by a normal distribution (blue). An exponential distribution (red) could indicate that chronic stress causes random reproductive failure among individuals in the population (A). A normal distribution (red) would suggest that subpopulations of individuals deploy qualitatively distinct reproductive strategies (B). Regardless of the explanation, there is a dichotomy of reproductive behaviors among individuals within populations under temperature stress.

The reproductive systems of individual *C. elegans* worms exhibited a heterogeneous response to temperature stress, manifested as more variable brood sizes. Several possible explanations can account for this phenomenon. Animals at higher temperatures might have an increased probability of a discrete failure event. This could plausibly give rise to two populations of animals—some reproducing at a relatively high rate, similar to (although slower than) that at lower temperatures—and some that have a *broken* reproductive system. Under this scenario, the combined distribution of brood sizes at a given temperature could be described as a mixture of a normal distribution, corresponding to robustly reproducing animals, and an exponential distribution, reflecting waiting time to a failure event (Figure 8A).

Alternatively, the observed heterogeneity could be indicative of a dichotomy of reproductive strategies (Figure 8B). Phenotypic switching—the responsive or stochastic shift between two discrete modes of behavior—has been shown to be an important part of adaptation to environmental stress in unicellular organisms [81], [82], [83]. Our results are consistent with the possibility that animals adopt aggressive or conservative strategies by altering the rates of oocyte development. At higher temperatures, more worms shift from aggressive (fast) to conservative (slow) egg-laying behavior. In our model, the primary difference between these populations is *k*_max_, the initial egg-laying rate before signal from sperm becomes rate limiting. It is conceivable that the observed heterogeneity could represent a bet-hedging approach in which some individuals in a population continue reproducing “expecting” conditions to become favorable soon, while others delay reproduction until conditions improve. Such a strategy may be advantageous for coping with the ever-changing environment [84].

Our results serve as a demonstration of the utility of macro-level modeling for understanding complex biological systems. We can envision the application of similar models to the understanding of other phenomena that involve mass transfer. Examples would include gas exchange in the respiratory system, filtration in the excretory system, and nutrient extraction in the intestine. More broadly, any system that consists of an orderly transition between defined compartments or states could be amenable to the type of analysis presented here. This would include development and tumorigenesis. Considerable, time-resolved experimental data are essential as are the knowledge of the initial conditions and the understanding of at least some interactions within the system. We believe that macro-level modeling can serve as a useful complement to more fine-grained approaches in the analysis of complex biological systems.

## Materials and Methods

### Strains

*Caenorhabditis elegans* Bristol wild-type N2, as well as *CB4419(tra-3(e2333))* [74], *FX544(cdc-48.1(tm544))* [75], *DA695(egl-19(ad695))* [79], and *YR27(egl-19(ad695)/tra-3(e2333))* mutants, were maintained at 20°C as described in Brenner [85]. *CB4419(tra-3(e2333))* is an allele of *tra-3* that is not temperature sensitive and does not affect the somatic gonad [67]. This allele causes a delay in the switch from spermatogenesis to oogenesis and a concomitant increase in the number of sperm. *FX544(cdc-48.1(tm544))* is a deletion mutant of a gene that regulates *tra-1*. In this mutant, the switch from spermatogenesis to oogenesis is premature and fewer sperm are produced [75]. *DA695(egl-19(ad695))* is a mutation in the α1 subunit of an L-type voltage-activated Ca^2+^ channel that causes myotonia and constitutive egg laying [86]. Mutant strains were obtained from the *Caenorhabditis* Genetics Center. To construct the double mutant, *YR27(egl-19(ad695)/tra-3(e2333))*, CB4419 males were mated to DA695 hermaphrodites. The progeny were allowed to self and double mutant candidates were selected on the basis of empty uterus and large brood size phenotypes. The genotype was confirmed by sequencing.

### Egg-laying experiments

To standardize the environment for nematode development, we prepared 60 mm NGM agar plates 48 to 62 h prior to experiments using 10 mL of medium per plate and seeded these plates with 100 µL of saturated OP50 culture 24 h before nematodes were transferred onto the plates. We prepared synchronized cultures of L1 larvae using hypochlorite treatment of gravid hermaphrodites [87]. The liberated eggs were left on a shaker for 18±3 h at room temperature (23–24°C) in M9 buffer—sufficiently long for the population to arrest at the L1 molt. The L1 larvae were placed onto the plate in contact with bacteria to synchronize the sensing of food and the termination of L1 diapause. This transfer of L1 larvae corresponds to 0 h in relation to L1 arrest and serves as the benchmark for timing in the rest of the experiments. The developing nematodes were maintained at 20°C and microscopic examination of worms at 44 h post-L1 arrest confirmed that more than 92% of nematodes were late-L4. Since a thin bacterial lawn with a small area increases both the density and visibility of laid eggs, we seeded new NGM plates with 5 µL of 1∶1000 dilution of saturated OP50 culture in Lysogeny broth (LB) 24±2 h after L1 arrest. We transferred single nematodes to the new NGM plates 1–2 h before the temperature shift.

The time designated for temperature shift was determined for each strain by enumerating eggs in the proximal gonad and fertilized embryos in the uterus. At 48, 50, 52 and 54 hours post L1 arrest, we examined twenty-five worms from each strain and counted the number of mature oocytes in the anterior and posterior gonad arms as well as the number of fertilized embryos in the uterus. Compared to N2, *FX544* and *CB4419* animals were delayed about three hours but otherwise appeared normal. The plates were moved into incubators at the experimental temperature shortly after the nematodes reached young adulthood: 48 h for N2, and 51 h post-L1 arrest for *FX544* and *CB4419* mutants. We measured temperature in each of the incubators with recording thermometers and discarded any time courses in which fluctuations were greater than 1°C.

We counted the total number of embryos on a plate manually using a dissecting microscope. We measured time courses at 2 h intervals for the first 12 h. For longer time courses at lower temperatures (20 and 25°C), we collected additional measurements every 12 h until egg-laying had ceased. To avoid unnecessary and possibly confounding temperature fluctuations for the time points recorded at 2 h intervals, we used new animals for each time point and discarded the plates after the number of eggs had been counted. To avoid the accumulation of offspring for time points recorded at 12 h increments, we removed the nematodes from the incubator, transferred them to new plates and returned them to their incubators within 10±5 minutes of their removal.

Experiments for each temperature were replicated on different days at least four times with at least one experiment in both the Morimoto and Ruvinsky laboratories. Thermometers between laboratories were within 0.1°C. Analysis of the individual trials suggests that small variations in developmental timing at the onset of stress contribute significantly to the variation in the total eggs laid.

### Viability and motility experiments

Populations of nematodes were synchronized as described above with the following notable exceptions: (i) the worms were not transferred onto new plates before exposure to stress conditions; (ii) we stressed populations of 20–40 animals instead of using plates with single nematodes; (iii) we seeded the plates used for developing worms with 5 µL of 1∶1000 dilution of saturated OP50 culture instead of saturated OP50 culture.

Viability and motility were assayed at 12 h increments by removing a different set of animals from the incubator at each time point, completing the measurements at room temperature, and discarding the worms. We touched animals with platinum wire to assess if they were motile or dead. Animals were scored as motile if they crawled at least one body length after gentle touch. Animals were scored as dead if they were unresponsive to touch and did not exhibit pharyngeal pumping.

These experiments were replicated on different days at least three times in the Ruvinsky Lab for each temperature. An average of 164 and 235 animals were used for each time point at 30 and 31°C, respectively. Time points were counted by multiple lab members to limit operator error.

### Egg retention experiments

Synchronized cultures of N2, CB4419, FX544 and DA695 were prepared and plated as for the egg-laying protocol described above. Twenty worms were singled for each temperature tested. At t = 0 (48 hours post L1 arrest for N2 and *DA695* and 51 hours post L1 arrest for *CB4419* and *FX544*), the twenty plates were shifted to 20, 25 or 28°C. After twenty-four hours of heat stress, the adult hermaphrodites were dissolved on the plate in 10 µL of alkaline hypochlorite solution and the eggs that had been retained in the worm were counted. Two trials were conducted for each strain.

### Statistical analysis

We used the permutation test [88], a bootstrapping procedure, to compare distributions of brood sizes (Figure 2) and coefficients of variation between brood sizes at different temperatures (Figure S4). For each comparison, the bootstrapping was repeated 10^6^ times. The estimated probability that the data could be observed, given the null model is, is the fraction of bootstrapped results that is *at least as extreme* as *d*.

## Supporting Information

Figure S1 Inter-lab results are no more different than intra-lab results.
(PDF)

Figure S2 At permissive temperatures, brood size distributions are normal throughout reproductive lifetime.
(PDF)

Figure S3 Brood sizes are normally distributed.
(PDF)

Figure S4 Coefficient of variation of brood sizes as a function of temperature.
(PDF)

Table S1 Summary of experiments performed to assess the effect of chronic temperature change on motility and viability.
(PDF)

Table S2 Summary of experiments performed with mutant *C. elegans* strains.
(PDF)

Table S3 Summary of egg retention experiments.
(PDF)

Text S1 Supporting text. The supporting text covers the verification of inter-lab experimental consistency, the evaluation of model assumptions, a description of simplifying assumptions, and a derivation of the gamete dynamics.
(PDF)
