# Supplementary material for: Macro-level Modeling of the Response of C. elegans Reproduction to Chronic Heat Stress
Source: PLoS Comput Biol. 2012 Jan 26;8(1):e1002338. doi: 10.1371/journal.pcbi.1002338 (PMC3266876; doi:10.1371/journal.pcbi.1002338)
Supplement: Figure S1 — Inter-lab results are no more different than intra-lab results. (PDF) [file pcbi.1002338.s001.pdf]

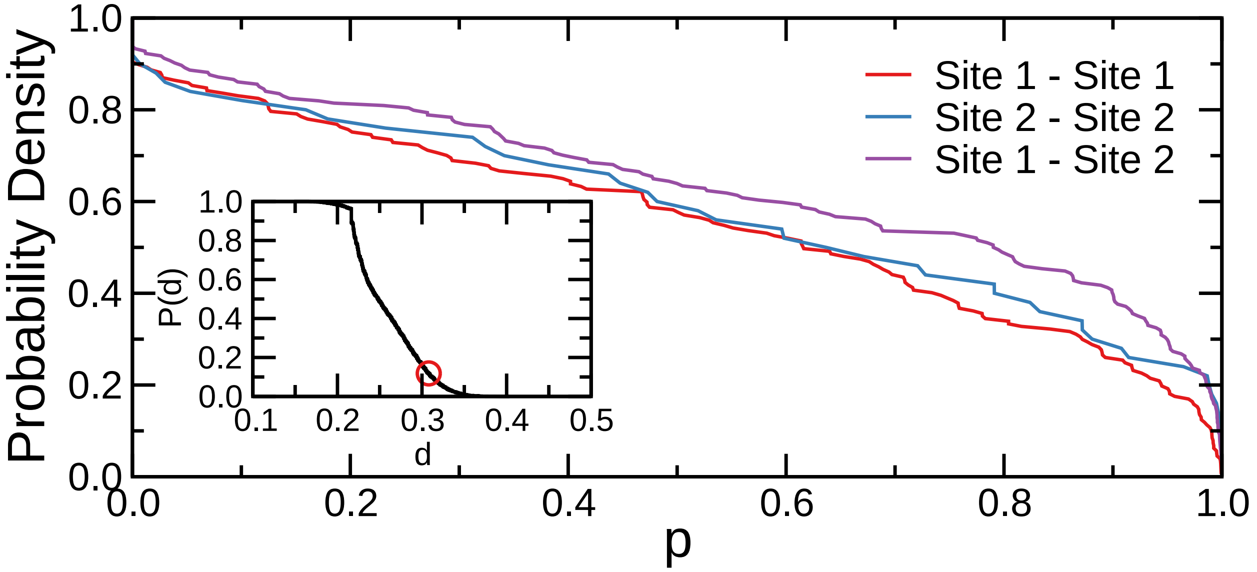

Figure S1: Inter-lab results are no more different than intra-lab results. Distributions of p-values from Kolmogorov-Smirnov tests comparing all pairs of time- and temperature-matched experiments. Intersite pairs (purple) are no more different than intrasite (red and blue) pairs ($p = 0.11 \pm 0.01$). A permutation test produces an ensemble of p-values that are consistent with the null hypothesis that differences among the intra- and inter-lab comparisons are indistinguishable (inset).
