# Supplementary material for: Macro-level Modeling of the Response of C. elegans Reproduction to Chronic Heat Stress
Source: PLoS Comput Biol. 2012 Jan 26;8(1):e1002338. doi: 10.1371/journal.pcbi.1002338 (PMC3266876; doi:10.1371/journal.pcbi.1002338)
Supplement: Figure S2 — At permissive temperatures, brood size distributions are normal throughout reproductive lifetime. (PDF) [file pcbi.1002338.s002.pdf]

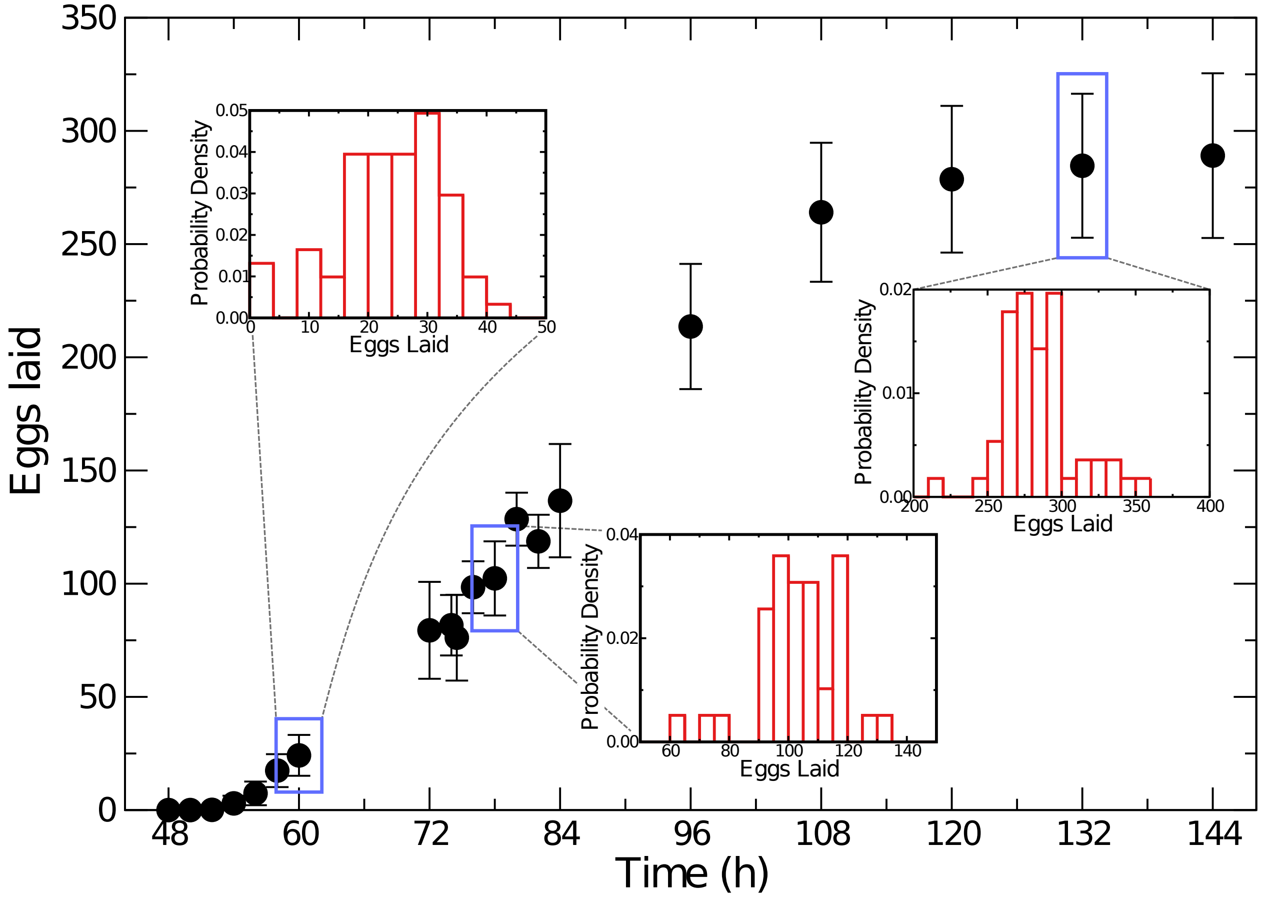

Figure S2: At permissive temperatures, brood size distributions are normal throughout reproductive lifetime.
