# Supplementary material for: Macro-level Modeling of the Response of C. elegans Reproduction to Chronic Heat Stress
Source: PLoS Comput Biol. 2012 Jan 26;8(1):e1002338. doi: 10.1371/journal.pcbi.1002338 (PMC3266876; doi:10.1371/journal.pcbi.1002338)
Supplement: Figure S3 — Brood sizes are normally distributed. (PDF) [file pcbi.1002338.s003.pdf]

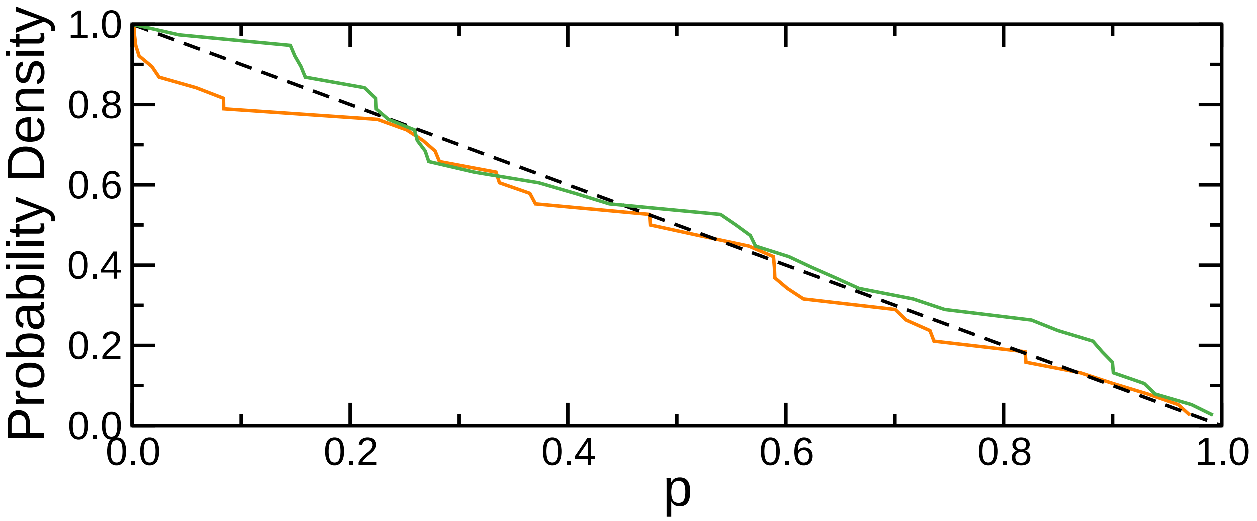

Figure S3: Brood sizes are normally distributed. Empirical distribution of p-values (orange), is consistent with the theoretical uniform distribution (black dashed). To confirm this, we generated 10,000 distributions consistent with the hypothesis that the empirical distribution is uniform. One of these is shown for illustration (green). Details of the experimental procedure are given in Supplemental Text.
