# Supplementary material for: Macro-level Modeling of the Response of C. elegans Reproduction to Chronic Heat Stress
Source: PLoS Comput Biol. 2012 Jan 26;8(1):e1002338. doi: 10.1371/journal.pcbi.1002338 (PMC3266876; doi:10.1371/journal.pcbi.1002338)
Supplement: Figure S4 — Coefficient of variation of brood sizes as a function of temperature. (PDF) [file pcbi.1002338.s004.pdf]

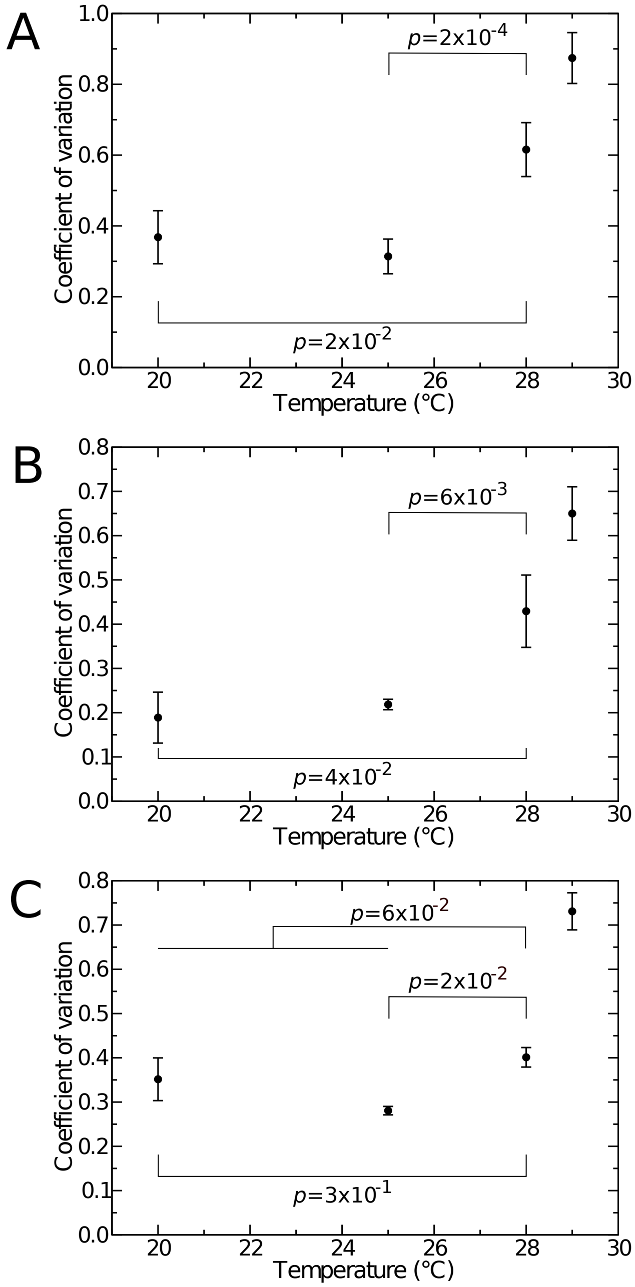

Figure S4: Coefficient of variation of brood sizes as a function of temperature. The coefficient of variation increases with temperature when data from all time points were considered (A), when only data collected at 72h were considered (B), and when brood size distributions with similar means (these data are boxed in Figure 2) were considered (C). p-values were computed using the permutation test described in Materials and Methods. Error bars represent $\pm$1SE.
