# Supplementary material for: Macro-level Modeling of the Response of C. elegans Reproduction to Chronic Heat Stress
Source: PLoS Comput Biol. 2012 Jan 26;8(1):e1002338. doi: 10.1371/journal.pcbi.1002338 (PMC3266876; doi:10.1371/journal.pcbi.1002338)
Supplement: Table S1 — Summary of experiments performed to assess the effect of chronic temperature change on motility and viability. (PDF) [file pcbi.1002338.s005.pdf]

Table S1: Summary of experiments performed to assess the effect of chronic temperature change on motility and viability.

| Temperature (°C) | Independent Experiments | Nematodes Assayed |
|---|---|---|
| 20 | 7 | 2,243 |
| 30 | 2 | 1,306 |
| 31 | 3 | 2,830 |
| | **12** | **6,379** |
