# Supplementary material for: Macro-level Modeling of the Response of C. elegans Reproduction to Chronic Heat Stress
Source: PLoS Comput Biol. 2012 Jan 26;8(1):e1002338. doi: 10.1371/journal.pcbi.1002338 (PMC3266876; doi:10.1371/journal.pcbi.1002338)
Supplement: Table S2 — Summary of experiments performed with mutant C. elegans strains. (PDF) [file pcbi.1002338.s006.pdf]

Table S2: Summary of experiments performed with mutant *C. elegans* strains.

Strain	Temperature (°C)	Independent Experiments	Nemotodes Assayed	Eggs Counted
tra-3	20	2	113	11,333
	25	2	124	11,575
	28	4	225	8,629
cdc-48.1	20	2	129	10,644
	25	2	97	4,747
	28	2	97	2,089
		**14**	**785**	**49,017**
