# Supplementary material for: Macro-level Modeling of the Response of C. elegans Reproduction to Chronic Heat Stress
Source: PLoS Comput Biol. 2012 Jan 26;8(1):e1002338. doi: 10.1371/journal.pcbi.1002338 (PMC3266876; doi:10.1371/journal.pcbi.1002338)
Supplement: Table S3 — Summary of egg retention experiments. (PDF) [file pcbi.1002338.s007.pdf]

Table S3: Summary of egg retention experiments.

| Strain | Temperature ($^{\circ}$C) | Nematodes Assayed | Eggs Counted |
|---|---|---|---|
| N2 | 20 | 147 | 7,225 |
| | 25 | 39 | 3,645 |
| | 28 | 39 | 1,620 |
| *tra-3* | 20 | 40 | 2,909 |
| | 25 | 36 | 4,014 |
| | 28 | 40 | 1,982 |
| *cdc-48.1* | 20 | 40 | 2,080 |
| | 25 | 39 | 2,499 |
| | 28 | 40 | 1,296 |
| *egl-19* | 20 | 39 | 2,554 |
| | 25 | 39 | 2,258 |
| | 28 | 40 | 2,287 |
| | | **578** | **34,369** |
