# Supplementary material for: Macro-level Modeling of the Response of C. elegans Reproduction to Chronic Heat Stress
Source: PLoS Comput Biol. 2012 Jan 26;8(1):e1002338. doi: 10.1371/journal.pcbi.1002338 (PMC3266876; doi:10.1371/journal.pcbi.1002338)
Supplement: Text S1 — Supporting text. The supporting text covers the verification of inter-lab experimental consistency, the evaluation of model assumptions, a description of simplifying assumptions, and a derivation of the gamete dynamics. (PDF) [file pcbi.1002338.s008.pdf]

Supplemental Text for

Macro-level modeling of the response of *C. elegans* reproduction to chronic heat stress

McMullen, *et al*.

Interlab experimental consistency

To ensure the robustness of the results we report, we performed temperature stress experiments in parallel at two laboratories: the Morimoto Lab at Northwestern University and the Ruvinsky Lab at the University of Chicago. Having established the calibration of temperature between the two sites, we investigated whether there were observable differences between the data collected at the Morimoto (Set 1) and Ruvinsky (Set 2) labs. To this end, we compared distributions of brood sizes, holding temperature and time constant. Any differences in the distributions must be accounted for by variation due to (a) differences across sites, (b) differences across experimental preparations, or (c) statistical fluctuations.

We compared the data from every pair of time- and temperature-matched samples by performing a permutation test [1]. Permutation tests are a form of bootstrapping used to compare sets of data when distributional assumptions of parametric tests are violated (see Materials and Methods). Though we will show below that the distribution of brood sizes over long times is normal (and therefore amenable to a Student's t test), this assumption is violated for time points early in the animals' reproductive life.

One can compute a Kolmogorov-Smirnov (KS) test statistic d for pair i of samples [2]. Because we make no distributional assumptions about the samples, we use resampling to test the null hypothesis that d is not significantly larger than one one would expect by sampling the pooled samples randomly. This yields a measure p reflecting the probability that this null hypothesis is true. Low values of p indicate support for rejecting the null hypothesis—an argument that the distributions are indeed different.

Because there are many pairs of samples, this constitutes a multiple testing problem [3, 4]. If the data are consistent with the null hypothesis, all values of p are equally likely. Indeed, one expects that the distribution of the ensemble of p-values $\{p\}$ are uniformly distributed. This is clearly not the case (Figure S1), suggesting that time- and temperature-matched samples are more different than would be explained by chance alone. However, this may be due partially to differences across experimental preparations. To isolate the variation due to inter-site differences, we computed the

KS test statistic for the distribution of $\{p\}$ for the inter-site paired samples. Using the same hypothesis testing scheme outlined above (Using the ensemble of all $\{p\}$ for sampling), we could not garner enough evidence to reject the null hypothesis (Figure S1, inset, $p = 0.11 \pm 0.01$). These data are consistent with the hypothesis that the differences in samples between sites are no larger than the differences within sites.

Evaluation of model assumptions

To confirm our assumption that the number of eggs laid by hermaphrodite worms is described by a Gaussian distribution, we tested the normality of the data generated by experiments performed in the Morimoto and Ruvinsky laboratories. For each experiment, we applied the Kolmogorov-Smirnov (KS) test to the data collected at each time point greater or equal to 72 hours for 20°C animals. This threshold time point is arbitrary, but sufficiently large to minimize any skewness due to counting statistics.

Using Monte Carlo hypothesis testing, the KS-statistic can be associated with a p-value for rejecting the null hypothesis that a distribution is Gaussian. Because we applied this test to 38 distributions, this presents a multiple testing problem. The p-value in this case also specifies a false-positive rate, so for a threshold of rejection at $p = 0.05$, treating these distributions as independent tests will cause us to incorrectly reject 5% of the null hypotheses, even if they all are consistent. To approach this problem we test the hypothesis that the p-values calculated from the KS-statistics for each of the data distributions themselves are distributed uniformly from 0 to 1 (Figure S3). Using Monte Carlo hypothesis testing, we cannot reject the hypothesis that this distribution is consistent with a uniform distribution ($p = 0.43 \pm 0.01$). This supports our assumption that the number of eggs laid is normally distributed.

Model simplification

In the text, we argued that some aspects of the model shown in Figure 3 (Equation 4) can be simplified without loss of descriptive power. From Equations 1 and 2, expressions for the fluxes F_g^o and F_o^o can be written,

$$F_g^o = k_g - k_s O \tag{S1}$$

$$F_o^o = k_o^* O S_a \, . \tag{S2}$$

Following from Figure 3B, the balance describing the time rate of change of number of oocytes is,

$$\frac{dO}{dt} = F_g^o - F_o^o\,. \tag{S3}$$

When oocyte development is sufficiently fast compared to downstream processes [5], oocyte development can be taken to be in *pseudo-steady-state*. That is, at the time scale we are interested in, $F_g^o \approx 0$. This approximation allows us to write an expression for the steady-state value O,

$$\begin{aligned} 0 &= F_g^o - F_o^o \\ &= k_g - k_s O - k_o^* O S_a && \text{(S4)} \\ O &= \frac{k_g}{k_s + k_o^* S_a}\,. && \text{(S5)} \end{aligned}$$

When k_s and k_g are relatively large ($k_s \gg k_o^* S_a$), the number of oocytes O depends weakly on S_a, and is essentially constant. Equation S2 thereby can be approximated as,

$$\begin{aligned} F_o^o &\approx k_o^* \frac{k_g}{k_s + k_o^* S_a} S_a && \text{(S6)} \\ &= k_o S_a\,, && \text{(S7)} \end{aligned}$$

yielding Equation 3, where the constant $k_o = k_o^* k_g / \left(k_s + k_o^* S_a\right) \approx k_o^* k_g / k_s$ is treated as a constant and as a free parameter in our model.

Derivation of gamete dynamics

Our model involves a number of coupled differential equations that describe the dynamics of various components of the system. It is possible to solve these equations explicitly, as described below. However, in practice, it is also useful to evaluate them numerically.

The time-evolution of the number S_a of active sperm can be expressed as

$$\frac{dS_a}{dt} = \max \begin{Bmatrix} -k^{max} - k_d S_a \\ -k_o S_a - k_d S_a \end{Bmatrix}\,. \tag{S8}$$

The number of active sperm is non-increasing over the lifetime of the animal. As such, late in the reproductive lifetime $k^{max} > k_o S_a$, and there exists a time τ at which $k^{max} = k_o S_a$. Therefore, Eq. S8 can be expressed,

$$\frac{dS_a}{dt} = \begin{cases} -k^{max} - k_d S_a & \text{if } t < \tau \text{, else} \\ -k_o S_a - k_d S_a \end{cases} . \tag{S9}$$

The evolution $S_a(t)$ for both of these cases is directly solvable, yielding,

$$S_a(t) = \begin{cases} -\frac{k^{max}}{k_d} + \left(\frac{k^{max}}{k_d} + S_a^0\right) e^{-k_d t} & \text{if } t < \tau \text{, else} \\ \frac{k^{max}}{k_o} e^{(k_o + k_d)(\tau - t)} \end{cases} , \tag{S10}$$

where S_a^0 is the number of sperm present at $t = 0$. At $t = \tau$, the two cases in Eq. S10 are equivalent, allowing us to solve explicitly for the value of τ,

$$\tau = \frac{1}{k_d} \log \left[\left(\frac{k^{max}}{k_d} + S_a^0 \right) \frac{k_o k_d}{k^{max}(k_o + k_d)} \right] . \tag{S11}$$

The differential equation describing the evolution of the number O_f of fertilized oocytes follows similar conditions as the system transitions between oocyte-limited and sperm-limited states,

$$\frac{dO_f}{dt} = \begin{cases} k^{max} & \text{if } t < \tau \text{, else} \\ k_o S_a \end{cases} . \tag{S12}$$

When $t < \tau$ the dynamics are trivial ($O_f(t) = k^{max} t$); however, the time-evolution after $t = \tau$ is dependent on the number of sperm, which complicates the derivation.

$$\begin{aligned} O_f(t) + C &= k_o \int dt\, S_a(t) \\ &= k_o \int dt\, \frac{k^{max}}{k_o} e^{(k_o + k_d)(\tau - t)} \\ &= k^{max} \int dt\, e^{-(k_o + k_d)(\tau - t)} \\ &= -\frac{k^{max}}{k_o + k_d} e^{(k_o + k_d)(\tau - t)} , \end{aligned} \tag{S13}$$

where C is a constant of integration. At $t = \tau$, the two cases in Eq. S12 are equivalent. As such, $O_f(t = \tau) = k^{max}\tau$.

$$\begin{aligned} k^{max}\tau + C &= -\frac{k^{max}}{k_o + k_d} e^{(k_o+k_d)(\tau-\tau)} \\ k^{max}\tau + C &= -\frac{k^{max}}{k_o + k_d} \\ C &= -\frac{k^{max}}{k_o + k_d} - k^{max}\tau \,. \end{aligned} \tag{S14}$$

Together, these equations describe the time-evolution of the observable O_f,

$$O_f(t) = \begin{cases} k^{max} t & \text{if } t < \tau \text{, else} \\ \frac{k^{max}}{k_o+k_d} + k^{max}\tau - \frac{k^{max}}{k_o+k_d} e^{(k_o+k_d)(\tau-t)} & \end{cases} , \tag{S15}$$

where τ is defined in Eq. S11.

References

[1] Efron B, Tibshirani RJ (1994) An Introduction to the Bootstrap (Chapman & Hall/CRC Monographs on Statistics & Applied Probability). Chapman and Hall/CRC, 1 edition.

[2] D'Agostino RB, Stephens MA (1986) Goodness-of-Fit Techniques. New York, NY: Marcel Kekker, Inc.

[3] Reiner A, Yekutieli D, Benjamini Y (2003) Identifying differentially expressed genes using false discovery rate controlling procedures. Bioinformatics 19: 368–375.

[4] Shaffer JP (1995) Multiple hypothesis testing. Annu Rev Psychol 46: 561–584.

[5] McCarter J, Bartlett B, Dang T, Schedl T (1999) On the control of oocyte meiotic maturation and ovulation in Caenorhabditis elegans. Dev Biol 205: 111–128.
